# Rosacea associated with increased risk of generalized anxiety disorder: a case-control study of prevalence and risk of anxiety in patients with rosacea^☆^^☆☆^

**DOI:** 10.1016/j.abd.2019.03.002

**Published:** 2019-10-26

**Authors:** Pinar Incel Uysal, Neslihan Akdogan, Yildiz Hayran, Ayse Oktem, Basak Yalcin

**Affiliations:** Dermatology Department, Ankara Numune Training and Research Hospital, Ankara, Turkey

**Keywords:** Anxiety, Anxiety disorders, Depression, Rosacea

## Abstract

**Background:**

Rosacea may result in emotional distress and anxiety. However, data on the presence of generalized anxiety disorder in rosacea patients are scarce.

**Objective:**

The aim of the study was to detect the frequency and level of anxiety and depression in patients with rosacea.

**Methods:**

A total of 194 consecutive rosacea patients and 194 age- and sex-matched controls were enrolled. Severity of rosacea was assessed in patients according to the criteria of the National Rosacea Society Ethics Committee. Both patients and controls were evaluated by the Generalized Anxiety Disorder 7-item scale, and severity was measured by the Generalized Anxiety Disorder-Adult.

**Results:**

Individuals who were diagnosed with an anxiety and/or depressive disorder were more common in patient group (24.7% *vs*. 7.2%, p < 0,01). Female patients were particularly at risk for having generalized anxiety disorder (OR = 2.8; 95% CI 1.15–7.37; *p* = 0.02).

**Study limitations:**

Single center study and limited sample size.

**Conclusions:**

Rosacea patients show greater risk of having anxiety disorders, including generalized anxiety disorder. Female patients, those with lower educational levels, those with phymatous subtype, untreated patients, and patients with prior psychiatric morbidity may be at particular risk for anxiety. It is essential to consider the psychological characteristics of patients to improve their well-being.

## Introduction

Rosacea is a chronic skin disorder characterized by inflammatory papules, telangiectasias, and facial erythema, which can result in psychosocial consequences and emotional distress. Remissions and exacerbations are major characteristics of the disease. Originally, four variants of rosacea were described by the National Rosacea Society Ethics Committee (NRSEC) as follows: erythematotelangiectatic, papulopustular, phymatous, and ocular form.^1^ In 2017, the NRSEC reported updated diagnostic criteria for rosacea.^2^ The committee has proposed a standard grading system for rosacea, a clinical scorecard for rosacea, which provides a practical and useful tool for clinical practice as well as research studies.^1^

Patients with rosacea are more likely to have depression, low self-esteem, social phobia, and stress.^3^, ^4^, ^5^ Depression is common among patients with rosacea and there is a direct relationship between rosacea severity and depression severity.^6^, ^7^ The persistent facial erythema of rosacea is caused by vasodilatation due to autonomic nerves or circulating vasoactive substances. Because there is no optimal and effective treatment modality for persistent redness, patients are prone to have decreased quality of life (QoL). Also, it has been shown that subjects with severe rosacea had worse mean Dermatology Life Quality Index (DLQI) scores than subjects with milder forms.^8^

A few studies have suggested that rosacea patients have increased risk of anxiety disorders.^7^, ^9^ Although the available evidence about the presence of anxiety disorders in patients with chronic dermatologic diseases including acne, psoriasis, and vitiligo is relatively clear in studies, this association has not been sufficiently addressed in rosacea. The present case–control study aimed to (i) estimate frequency of co-morbid depression and anxiety among rosacea patients, (ii) identify grade of anxiety in rosacea patients by the Generalized Anxiety Disorder 7-item scale (GAD-7) and severity measured by the Generalized Anxiety Disorder-Adult scale (GAD-Adult); (iii) to determine whether there is a relation between rosacea grade and anxiety grade.

## Methods

### Study design and patients

This was a cross-sectional, single-center case–control study with prospective recruitment of patients and matched control subjects in a tertiary clinic. Consecutive patients who were diagnosed with rosacea during the period between January 2017 and December 2017 were included in the study. There were 194 patients and age- and sex-matched controls included in this study. The control group consisted of healthy volunteers who presented with cosmetic complaints.

All participants were 18 years of age or older. All cases were subjected to systemic and dermatological examination. Exclusion criteria for the study and control group were as follows: age younger than 18, and patients having chronic systemic illnesses, chronic inflammatory dermatological diseases, or cognitive impairment.

### Data collection and measurements

Demographical and clinical data of the subjects were recorded. All patients with rosacea were examined by the same dermatologist and classified into four subtypes according to the standard NRSEC classification criteria.^1^ The clinical scorecard consisted of primary and secondary symptoms of patients (flushing, nontransient erythema, telangiectasia, burning/stinging, plaques, dryness, edema), and The physician's global assessment (IGA) and the patient's global assessment (PGA). Scores from 0 to 3 are assigned to each category and all scores are summed to obtain a single total score (0–48). Rosacea severity scores of all patients were recorded by the same dermatologist as absent, mild, moderate, or severe. Smoking and drinking habits were also noted. The rosacea clinical scorecard (available at https://www.rosacea.org/physicians/scoreindex.php) was completed for each patient. Patients and control subjects self-reporting any symptoms or previous clinical diagnosis of depression and/or anxiety were recorded. All patients were asked about presence of exacerbation or triggering of rosacea lesions with psychological stress. The medical history of subjects was also noted.

The GAD-7 scale is a validated screening tool and a measure for screening anxiety disorders including generalized anxiety disorder, panic disorder, and social anxiety disorder in the general population.^10^, ^11^ Total GAD-7 scores are presented from 0 to 21. The total score is a guide to assess severity of the anxiety as following: <5 mild anxiety; 5–10 moderate anxiety; >10 severe anxiety. In addition, the severity measure of the GAD-Adult is a ten-item measure to be completed on a scale of 0 (never) to 4 (all of the time) for assessing the severity of anxiety symptoms in individuals aged 18 and older.^12^ Each item asks the severity of symptoms during past seven days. The raw total score can range from 0 to 40. The average total score is a five-point scale, which allows to clinician to justify the severity of the anxiety disorder of the individual as follows: none (0), mild (1), moderate (2), severe (3), or extreme (4). The GAD-7 questionnaire and the severity measure of the GAD-Adult were developed based on Diagnostic and Statistical Manual of Mental Disorders (DSM) IV and V criteria, respectively.

All patients and control subjects completed the GAD-7 and the severity measure of the GAD-Adult (Table 1) to screen for anxiety disorders. Data of the patients who had repetitive admissions were included only once. Incomplete questionnaires or patients with incomplete data were excluded.

**Table 1:** Comparison of demographic characteristics of the study and control groups.

	Patients (*n* = 194)	Controls (*n* = 194)	*p*-Value
*Sex, n (%)*			
Female	147 (75%)	147 (75%)	–
Male	47 (25%)	47 (25%)	–

*Age, median (IQR) (years)*	47 (40–56)	46 (39–54.25)	–

*Habit of cigarette smoking, n (%)*			
Current smoker	41 (21.1%)	58 (29.9%)	p = 0.005
Ex-smoker	22 (11.3%)	7 (3.6%)	p = 0.005
Never smoked	131 (67.5%)	129 (66.5%)	p > 0.05

*Pack-years of cigarette smoking (mean* ± *SD)*	15.5 ± 1.2	12.4 ± 0.9	p > 0.05

*Alcohol consumption, n (%)*			p > 0.05
Ever used	172 (88.7%)	160 (82.4%)	
Last week	16 (8.2%)	17 (8.7%)	
Last month	6 (3%)	17 (8.7%)	

*Educational level, n (%)*			p < 0.001
Elementary school	97 (50%)	30 (15.4%)	
Middle school	24 (12.3%)	50 (25.7%)	
High school	46 (23.7%)	69 (35.5%)	
University	10 (5.1%)	37 (19%)	
No education	17 (8.7%)	8 (4.1%)	

IQR, interquartile range; SD, standard deviation.

### Statistics

Statistical analyses were carried out using SPSS software (v. 21.0 for Windows; SPSS Inc., Chicago, IL, United States). Parametric variables were expressed as means and standard deviations, and nonparametric variables were presented as medians and interquartile ranges. For categorical variables, the number of cases and percentages were used. The Kolmogorov–Smirnov test and histogram analyses were used to determine whether continuous variables were normally distributed. Normally distributed numeric variables were analyzed by Student's *t*-test and ANOVA. The chi-squared test or Fischer's exact test were used for analyzing categorical variables. Mann–Whitney *U* and Kruskal–Wallis tests were performed for comparing non-normally distributed numeric variables. Correlations of numeric variables were assessed by Spearman and Pearson tests. Multiple logistic regression models were created to examine the relationships between study variables and the recorded psychiatric diagnosis and presence of generalized anxiety disorder, providing odds ratios (OR) and 95% confidence intervals (95% CI). The level of significance was set as *p* < 0.05.

## Results

### Participant characteristics

The study enrolled 194 patients (147 female, 47 male) and 194 age- and sex-matched rosacea-free controls (147 female, 47 male). The median age of cases was 47 (ranging between 18 and 74). Ex-smokers were more common among the patient group. Alcohol intake (*p* = 0.083) and cigarette consumption (*p* = 0.59) were comparable between cases and controls. Having graduated from high school or university was more common among control subjects (*p* < 0.001). The median value of educational level was elementary school among patients, whereas it was high school in controls.

Demographical and life style characteristics and medical history of study and control groups are summarized in Table 1.

### Rosacea characteristics

The erythematotelangiectatic subtype was the most frequent (58.9%) subtype, and the disease severity was mild and moderate in most patients (85.1%). The median rosacea severity score of the patients was 12 (IQR: 10–16), which corresponds to the mild form of rosacea. Clinical characteristics and variables of clinical scorecard are shown in Table 2. A significant positive correlation was found among severity scores, PGA (*r* = 0.559; *p* < 0.001), and the physician rating by subtype (*r* = 0.805; *p* < 0.001).

**Table 2:** Disease characteristics of the patients with rosacea (*n* = 194).

Clinical characteristics of the patients	*n* = 194
*Disease subtypes*^a^, *n (%)*	
Erythematotelangiectatic	114 (58.7%)
Papulopustular	95 (48.9%)
Phymatous	9 (4.6%)
Ocular	10 (5%)
*Duration of the disease, median (IQR) (months)*	36 (12–72)

*Treatment modalities, n (%)*	
None	43 (22.2%)
Sun protection	16 (8.2%)
Topical treatments	82 (42.3%)
Combination of topical and oral antibiotics	37 (19%)
Oral isotretinoin	16 (8.2%)

*Physicians rating by subtype, n (%)*	
Mild	133 (68.5%)
Moderate	56 (28.9%)
Severe	5 (2.6%)

*Patient's global assessment, n (%)*	
Mild	64 (33%)
Moderate	89 (45.9%)
Severe	41 (21%)

*Grade of rosacea*^b^, *n (%)*	
Mild	77 (39.7%)
Moderate	88 (45.4%)
Severe	29 (14.9%)

*Severity score, median (IQR)*	12 (10–16)

IQR, interquartile range; SD, standard deviation.

a Some patients presented with mixed forms of disease.

b Mild group (range: 0–14), moderate group (range: 15–24), severe group (≥25).

### Rosacea and psychosocial disorders

Rosacea and associated psychosocial variables are presented in Table 3.

**Table 3:** GAD 7-item scale and GAD-Adult score, severity of anxiety, and personal history regarding psychosocial disorders of rosacea patients and control subjects.

	Patients (*n* = 194)	Controls (*n* = 194)	*p*-Value
GAD-7 score, median (IQR)	6 (3–10)	2 (0–3)	<0.001
GAD-Adult total score, median (IQR)	12 (7–17)	3 (1–8)	<0.001
GAD-Adult average total score, median (IQR)	1.2 (0.7–1.7)	0.3 (0.1–0.8)	<0.001
Severity of anxiety, median (IQR)	1 (1–2)	0 (0–1)	<0.001
Prior history of psychiatric morbidity, *n* (%)	48 (24.7%)	14 (7.2%)	<0.001
Anxiety disorder	21 (10.8%)	5 (2.6%)	p < 0.001
Depression	27 (12.9%)	9 (4.1%)	p = 0.004
Use of psychiatric drug at the time of data collection, *n* (%)	28 (14.4%)	10 (5.2%)	p = 0.002

GAD, generalized anxiety disorder; IQR, interquartile range; SD, standard deviation.

Personal history of previous diagnosis of an anxiety disorder and/or depression was more frequent among patient group (24.7% *vs.* 7.2%; *p* < 0.001). The authors found a significant difference in frequency of use of medication prescribed for any psychosocial disorder between patients and controls at the time of admission (14.4% in patients *vs.* 5.2% in controls; *p* = 0.002). In total, 3.6% of patients had both anxiety and depressive disorders, whereas no one in control group had prior history of both disorders. Patients with rosacea were more likely to have anxiety disorders (OR = 4.59; 95% CI: 1.69–12.43; *p* = 0.003) and depression (OR = 3.041; 95% CI: 1.38–6.07; *p* = 0.006) (Table 3).

The median values of GAD-7 (*p* < 0.001), the severity measure of GAD-Adult average (*p* < 0.001), and raw total scores (*p* < 0.001) were higher in patients than controls (Table 3). It was observed that 25% of patients (*n* = 50) screened positive (GAD-7 > 9) for GAD compared with 4.1% of controls (*n* = 8; *p* < 0.05). Patients with rosacea were likely to have higher grade of anxiety than control subjects.

There were no correlations between PGA, physicians rating by subtype (IGA) and anxiety scores (PGA and GAD total score: *p* = 0.95, *r* = 0.004; PGA and GAD average total score: *p* = 0.95, *r* = 0.004; PGA and GAD-7: *p* = 0.61, *r* = 0.037; PGA and severity of anxiety: *p* = 0.97, *r* = −0.003; IGA and GAD total score: *p* = 0.17, *r* = −0.099; IGA and GAD average total score: *p* = 0.25, *r* = −0.83; IGA and GAD-7: *p* = 0.45, *r* = −0.055; IGA and severity of anxiety: *p* = 0.32, *r* = −0.072).

### Risk factors for anxiety

Median scores of the GAD-7 and the severity measure of the GAD-Adult were comparable in erythematotelangiectatic and papulopustular types (*p* > 0.05). However, phymatous rosacea patients represented higher levels of all anxiety scores (*p* < 0.05). Female patients had higher anxiety scores and grades of anxiety severity than male patients (*p* < 0.05). Additionally, patients with prior history of psychiatric morbidity (anxiety disorder and/or depression) had higher scores than the others (*p* < 0.05). In comparison with others, patients who had reported flaring rosacea symptoms in response to psychological stress represented higher grades of anxiety severity (*p* < 0.001 for the GAD-7, GAD-Adult average scores, GAD raw total scores, and anxiety severity).

Patients with low educational levels had higher anxiety scores than patients with higher educational levels (GAD-7, GAD-Adult, and severity of anxiety) (*p* < 0.05). There was a negative correlation between anxiety scores and educational levels of patients (GAD-7: *p* = 0.014, *r* = −0.13; GAD-Adult total score: *p* = 0.018; *r* = −0.12; GAD-Adult average score: *p* = 0.018, *r* = −0.12; anxiety severity: *p* = 0.019, *r* = −0.12). The proportions of the patients receiving different types of treatment were similar in terms of anxiety scores (*p* > 0.05). However, the untreated patients were at risk of having severe anxiety (OR = 2.07; 95% CI: 1.43–4.06; *p* = 0.04). There were no significant relationships between age, age of disease onset, disease duration, smoking, or drinking habits and presence or severity of anxiety.

Risk of GAD (GAD-7 item score > 9) was significantly increased in females (OR = 2.8; 95% CI: 1.15–7.37; *p* = 0.02). There were no significant relationship between age, age of onset, educational status, disease duration, or treatment groups and presence of GAD (*p* = 0.18, *p* = 0.49, *p* = 0.65, and *p* = 0.22, respectively).

## Discussion

Studies have shown that psychiatric comorbidities and undetected psychopathologies can greatly impact the QoL of patients with dermatological disorders. Furthermore, these conditions may contribute to the clinical severity of skin disorders. Rosacea is one of the psychosomatic skin conditions that can fluctuate in accordance with emotional state. According to the most accepted consensus in the literature, in case of emotional stress, the neuro-immuno-cutaneous system (NICS) is responsible for releasing cytokines, mediators, and neurotransmitters contributing this complex interaction.^13^ It has already been reported that patients with rosacea have higher incidences of embarrassment, social anxiety, low-self esteem, and decreased DLQI.^14^ Besides feelings of stigmatization, all these conditions may lead to depression or anxiety disorders.^15^

In the present study, past or recent history of psychiatric illness and use of psychiatric medication were three to four times higher in patients than controls. Of the 194 patients, 3.4% had been previously diagnosed with both anxiety disorder and depression. Obviously, patients were at higher risk of having past or recent diagnosis of depression or anxiety. In line with other studies, rosacea increased the risk of both depression and anxiety.^7^, ^9^ The present study demonstrated higher GAD-7, GAD-Adult raw total and average scores, and higher grade of severity of anxiety in rosacea patients in comparison with control subjects. Furthermore, a considerable number of patients (25%, *n* = 50) had probable generalized anxiety disorder shown by the GAD-7. Many of the studies addressing anxiety have mainly focused on social anxiety, and have used QoL and/or Hospital Anxiety and Depression Scale (HDAS) measurements.^9^, ^16^, ^17^ In the literature search, the authors could not find a study using the GAD-7 and the GAD-Adult questionnaires in rosacea patients. Thus, the present study provides these valid scales for measuring anxiety grade and detecting generalized anxiety disorder in rosacea patients for the first time.

Alinia et al. reported a direct relationship between severity of rosacea and degree of depression.^18^ In contrast, the present study did not detect a correlation between anxiety scores or degree of anxiety and rosacea clinical score, PGA, and IGA. However, patients with phymatous rosacea had higher scores than the other subtypes. This result support previous reports suggesting that phymatous rosacea causes feelings of stigmatization and more overall QoL impairment.^16^, ^17^ It has been suggested that because of men tend to experience more severe forms of rosacea than women, they are more prone to have social anxiety and depression.^15^ Some studies discovered that males were more negatively affected by the disease than females.^16^, ^19^ However, in the present study, in the GAD-7 and the severity of measure of the GAD-Adult, female patients had significantly higher anxiety scores. Gender-related differences in the present study were consistent with some reports in the literature.^7^ Of note, these findings may be clearly explained with the fact that female patients seem to focus on the impact of rosacea on their appearance. Previous studies revealed that younger patients were more likely to be affected by rosacea.^16^, ^20^ However, the present study found no correlation between age and risk of depression or anxiety. It has been shown that low socio-economical status is associated with higher risk of depression.^7^ In this context, it is noteworthy to consider that negative correlation was present between anxiety scores and educational status. Regarding these results, patients with higher educational levels seem to be compliant with medications. This may improve their well-being and result in reduced anxiety.

It has been shown that effective treatment of the symptoms of rosacea leads significant improvement of psychosocial symptoms and health-related QoL.^17^, ^19^ In fact, any treatments targeting blushing help to alleviate depressive symptoms and social anxiety.^5^ Similarly, the present study found increased risk of anxiety in patients who had not been treated previously. These findings can be explained by the fact that of the 43 untreated patients, 81% attended for the first time, whereas the remaining were return patients.

Importantly, this study confirmed that female patients with rosacea are particularly at risk of GAD. It was observed that 84% (42/50) of these patients had not been referred to a psychiatry clinic before. Detection of high numbers of probable, previously undetected GAD cases among rosacea patients was another interesting finding of the present study. It must be remembered that the anxiety tools used in this study are unable to detect causality. Thereby, based on the results of this study, it is hard to identify whether anxiety could be a direct consequence of rosacea or a coincidental finding.

The large sample size and the use of validated tools are the main strengths of this study. However, this study has some limitations. The study population may not be representative of patients in the general population. Nonetheless, this hospital provides care to a large percentage and a wide range of socio-economic classes who belong to the surrounding community.

## Conclusion

These data suggest that rosacea patients appear to have anxiety independent of their disease severity. Some patient groups may be at greater risk of having anxiety. Screening patients with QoL tools may not reflect exact psychopathology. The GAD-7 and the severity measure of the GAD-Adult are valid and easy-to-apply tools to detect patients at risk in daily clinical practice. Physicians should be aware of this relationship in order to provide psychological support as part of the psychosomatic treatment strategy.

## Ethical approval

Written consent was obtained from all studied patients and control subjects before the collection of data and questionnaires. The study protocol was approved by the University of Health Sciences, Ankara Numune Training and Research Hospital Ethics Committee of Clinical Studies (E-16-1064). The study protocol was in accordance with the Helsinki Declaration of 1975.

## Financial support

None declared.

## Author's contributions

Pınar Incel Uysal: Approval of the final version of the manuscript; conception and planning of the study; elaboration and writing of the manuscript; obtaining, analyzing and interpreting the data; effective participation in research orientation; intellectual participation in propaedeutic and/or therapeutic conduct of the cases studied; critical review of the literature; critical review of the manuscript.

Neslihan Akdogan: Approval of the final version of the manuscript; obtaining, analyzing and interpreting the data; effective participation in research orientation; intellectual participation in propaedeutic and/or therapeutic conduct of the cases studied; critical review of the literature

Yildiz Hayran: Statistical analysis; approval of the final version of the manuscript; elaboration and writing of the manuscript; effective participation in research orientation; intellectual participation in propaedeutic and/or therapeutic conduct of the cases studied; critical review of the literature.

Ayse Oktem: Approval of the final version of the manuscript; conception and planning of the study; obtaining, analyzing and interpreting the data; effective participation in research orientation; critical review of the manuscript.

Basak Yalcin: Approval of the final version of the manuscript; conception and planning of the study; effective participation in research orientation; intellectual participation in propaedeutic and/or therapeutic conduct of the cases studied; critical review of the manuscript.

## Conflicts of interest

None declared.
